# Emission Factors of CO_2_ and Airborne Pollutants and Toxicological Potency of Biofuels for Airplane Transport: A Preliminary Assessment

**DOI:** 10.3390/toxics10100617

**Published:** 2022-10-18

**Authors:** Maurizio Gualtieri, Massimo Berico, Maria Giuseppa Grollino, Giuseppe Cremona, Teresa La Torretta, Antonella Malaguti, Ettore Petralia, Milena Stracquadanio, Massimo Santoro, Barbara Benassi, Antonio Piersanti, Andrea Chiappa, Manuele Bernabei, Gabriele Zanini

**Affiliations:** 1 ENEA, Division of Models and Technologies for Risk Reduction, Via Martiri di Monte Sole 4, 40146 Bologna, Italy; 2 ENEA, Division of Health Protection Technologies, Via Anguillarese, 301, 00123 Rome, Italy; 3 Italian Air Force, Aerospatial Testing Division, Aerospace Materials and Technology Department, Aeroporto Militare de Bernardi 00071 Pratica di Mare, Pomezia, 00040 Rome, Italy

**Keywords:** biofuels, emission factors, in vitro exposure, hazard assessment, human exposure, aviation, climate change, air pollution

## Abstract

Aviation is one of the sectors affecting climate change, and concerns have been raised over the increase in the number of flights all over the world. To reduce the climate impact, efforts have been dedicated to introducing biofuel blends as alternatives to fossil fuels. Here, we report environmentally relevant data on the emission factors of biofuel/fossil fuel blends (from 13 to 17% *v*/*v*). Moreover, in vitro direct exposure of human bronchial epithelial cells to the emissions was studied to determine their potential intrinsic hazard and to outline relevant lung doses. The results show that the tested biofuel blends do not reduce the emissions of particles and other chemical species compared to the fossil fuel. The blends do reduce the elemental carbon (less than 40%) and total volatile organic compounds (less than 30%) compared to fossil fuel emissions. The toxicological outcomes show an increase in oxidative cellular response after only 40 min of exposure, with biofuels causing a lower response compared to fossil fuels, and lung-deposited doses show differences among the fuels tested. The data reported provide evidence of the possibility to reduce the climate impact of the aviation sector and contribute to the risk assessment of biofuels for aviation.

## 1. Introduction

Climate change is affecting the lives of populations all over the world. Expected increases in temperature are likely to be associated with increased mortality [1]. Furthermore, the environmental impacts of climate change on biodiversity, crop production, and animal habitats pose a serious threat to the planet as we know it [2,3,4].

To avoid such detrimental effects, governments have renewed their commitment by signing the Paris Agreement ([5]) which aims at limiting “global warming to well below 2, preferably to 1.5 degrees Celsius, compared to pre-industrial levels”. To achieve this goal, countries have agreed to reduce the emission of greenhouse gases and the extraction and consumption of fossil fuels.

The EU target to achieve carbon neutrality by 2050 requires action in several life, production, and societal sectors [6,7], with transport playing a relevant role [8]. Among the different sectors that use fossil fuels, aviation is the one which, in recent years, experienced the fastest growing rate [9]. Despite the improvements achieved in the last twenty years to increase fuel efficiency, the aviation sector has faced a tremendous increase in the number of flights and passengers, becoming the EU’s second largest greenhouse gas (GHG) emitter in the transport sector after road transport; in fact, the International Civil Aviation Organization (ICAO) estimated a potential three-fold increase in GHG emissions from the aviation sector from 2015 to 2050 [10].

Consequently, the EU have attempted (https://ec.europa.eu/clima/eu-action/transport-emissions/reducing-emissions-aviation_en accessed on 15 September 2022) to reduce the impact of this sector on climate change, acting at both the European and international levels. 

Besides the emission of carbon dioxide (CO_2_), aircrafts emit nitrogen oxides, soot, sulphate particles, and water [11]. These species influence cloudiness in the high atmosphere and hence solar radiation warming potential [12] and play a role in aerosol and ozone pollution in the troposphere, including at ground level [13]. 

To reduce the impact of aviation on the climate, great effort is being devoted to developing and testing fuel from renewable (therefore carbon-neutral) sources to substitute, partially or totally, the fossil fuels presently used [14,15]. Several different biofuels are currently available on the market, but hydroprocessed fatty acid esters and free fatty acid (HEFA) are considered the most promising for improving aviation carbon neutrality, although with some potential limitations to be considered [16]. Recently, the strict interplay between GHG emissions during fuel combustion and the energy demand of biofuel production has been reported, providing additional insight into the importance of considering the whole life cycle of the biofuel [17] when aiming at reducing production and emission costs. Laboratory tests conducted on different biofuel blends showed the capability of these fuels to reduce particle [18], CO, and NO_x_ [19] emissions. Similarly, a previous study [20] showed significant improvement in emissions with biofuel blends, findings confirmed also by inflight measurements. Notably, the most recent environmental report from the European Union Aviation Safety Agency (EASA [21]) clearly states that “European citizens are becoming increasingly aware of the affect that aviation activities have on their quality of life through climate change, noise and air quality...”. 

Therefore, besides reducing climate change, the health risks associated with the replacement of conventional fuels with bio-based fuels should be considered. In fact, while diesel exhaust from on-road engines is a carcinogen for humans [22], less information is available for aircraft emissions and even less for the exhaust from biofuel for aviation. Several authors have reported an increase in air pollution near airports. Nanoparticle concentrations close to airports were higher than the concentrations recorded downtown near a major road [23], with relative increases as high as 28-fold near some airports [24]. Interestingly, Pirhadi et al. reported that the main activities influencing the air quality surrounding airports were take-off and landing [25]. Moreover, airport workers are particularly exposed to engine exhaust and, depending on the kind of task, ground workers are usually exposed to higher ultrafine particle concentrations [26] and experience higher lung-deposited doses than office or security workers [27]. Moreover, the emissions from airports strongly affects the populations living nearby, and significant correlations have been found between the onset of health issues and residence in the proximity of airports [28,29]. In agreement, toxicological evaluations of jet fuel vapors showed a relevant impact on the immune system [30], while in vitro studies of sampled airport emissions underlined in vitro effects comparable to diesel exhaust [31,32].

Surprisingly, in view of these data there is still a lack of information on the emission factors and the potential toxicological effects and human dosimetry of the emissions from aircraft engines run with biofuel blends. This assessment is pivotal to avoid contrasting offsets between climate change reduction requirements and air pollution and human protection needs. In this manuscript, we therefore aim at discussing this major aspect by accounting for the emission indexes and the toxicological impacts of exhaust from an airplane fueled with biofuel blends and a fossil fuel. We measured the emissions of the Rolls-Royce Spey engine, which is a low-bypass turbofan engine, installed on an AMX “Ghibli”, a military ground attack jet, provided by the Italian Air Force. The aircraft was anchored to the ground and run with Jet A-1 synthetic fuel or different blends (13 and 17% HEFA in Jet A-1 fuel v/v, according to the safety requirements for the Air Force jet and personnel). The emissions were collected after preliminary mixing with the atmosphere and characterized for their physical and chemical properties. Moreover, the toxicological effects on BEAS-2B bronchial epithelial cells—exposed at the air–liquid interface to the emitted exhaust—and the human lung deposition, using the MPPD model, were determined to account for the potential hazard and the human exposure doses of the different fuels, thereby providing preliminary data for a risk assessment of the exhaust from different aviation fuels.

## 2. Materials and Methods

### 2.1. Sampling Line

**Figure 1:**
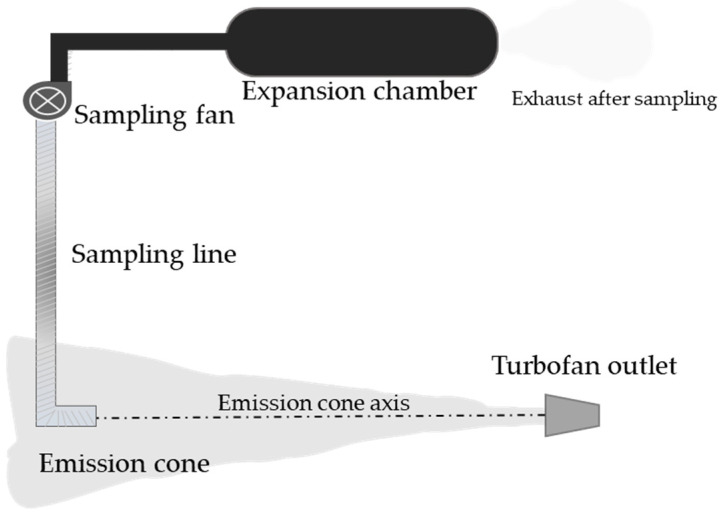
Schematic representation of the sampling line adopted to characterize the emissions from the Spey Mk 202 engine. The sampling inlet was placed in line with the axis of the exhaust emission cone. The suction of the emissions was performed by a sampling fan allowing a quasi-isokinetic sampling. The turbofan exhaust was then allowed to expand into an expansion chamber where instrument sampling lines were placed. The non-sampled exhaust was left to exit the expansion chamber during the whole sampling period.

Combustion emissions from an AMX fighter jet of the Italian Air Force equipped with a Spey engine (view Table 1 for more details) and fixed to the ground by means of a safety hook to avoid movement were collected at circa 20 m from the exhaust of the jet’s Rolls-Royce Spey turbofan (Figure 1). The sampling line for the exhaust was composed of an aluminum pipe (diameter of 20 cm and length of 20 m), with the inlet placed at 110 cm from the ground, connected to a tubular expansion chamber of 6 m^3^ (diameter 120 cm, length 5 m). The expansion chamber was made of conductive antistatic polyethylene to avoid particle losses. The expansion chamber was then fixed to the roof of the Transportable Laboratory MINNI by means of a PVC net to avoid any possible movement by local winds. The emissions were sampled isokinetically using a controlled fan at the end of the sampling line (Figure 1). Four different sampling lines were placed in the expansion chamber and connected to the online monitors and instruments located in the transportable laboratory. Two consecutive experiments were performed with a fossil fuel (Jet A-1 type) and four consecutive tests after adding to the fossil fuel (Jet A-1 type) a selected quantity of a hydroprocessed fatty acid esters and free fatty acid (HEFA) biofuel (according to the specifics of Annex II ASTM D 7566). The final concentration of the HEFA fuel ranged between 13% (first and second tests) and 17% (third and fourth tests; chemical analysis of this blend is reported in Appendix B Table A1) *v*/*v* in the fossil fuel. Two different thrusts were used during each fuel test, 50% and 70% (±5%) of the maximal nominal power, while the final emission test (4th test with 17% biofuel) was performed at a higher thrust (60 to 95%) to empty the tank of the biofuel blend. The shift from the lower to the higher thrust during each test was announced by an acoustic signal and recorded for subsequent interpretation of the data.

### 2.2. Emission Characterization

Emissions originating from fossil or biofuel combustion were sampled in the expansion chamber and were characterized according to the following online monitors: total volatile non-methane organic compounds monitor (TNMHC, Synspec ALPHA 115); SO_2_ monitor (TELEDYNE 101E H_2_S/SO_2_ Analyzer); NO_2_/NO/NO_x_ monitor (2B Technologies Model 405 nm); CO_2_ NDIR monitor (GO_3_ Project Monitor O_3_/CO_2_); Scanning Mobility Particle Sizer, SMPS (TSI 3938 SMPS); Optical Particles Counter (OPC GRIMM mod. 1.107) for particle size distribution from 250 nm to 1.7 µm; SUNSET Semi-Continuous Carbon Aerosol Analyzer, with denuder to remove volatile organic compounds (for measuring total carbon—TC, organic carbon—OC, and elemental carbon—EC) according to the NIOSH-like thermal protocol; Aerosol Chemical Speciation Monitor (ACSM) for the detection in PM_1_ of total organic matter (OA), ammonia (NH_4_^+^), sulphate (SO_4_^2−^), nitrate (NO_3_^−^), and chloride (Cl^−^); Cultex^®^ RFS Compact Type II module (CULTEX^®^, Germany) for the direct exposure of in vitro lung models. 

Besides the online monitors, three filter holders, connected to sampling pumps (Digit model, Zambelli, Italy, constant flow rate of 12.78 L/min), were used to collect exhaust samples for offline laboratory analyses. Pumps were switched on at the beginning of each test and switched off at the end to sample the separated test emissions, and a cyclone was used to select particles below PM_2.5_. One filter holder was used to sample the exhaust on PTFE filters (Pall Teflon with ring pore size 1.0 µm and diameter 47 mm), and the two other filter holders were used to sample the exhaust on quartz filters (Pall 2500-QUAT-UP, diameter 47 mm). The PTFE filters were used to quantify the sampled mass by gravimetric determination and the metal and the trace element by XRF technique (ED-XRF, Rigaku NEX CG); one quartz filter was used to determine the amount of soluble ions (Cl^−^, NO_3_^−^, SO_4_^2−^, Na^+^, K^+^, NH_4_^+^, Ca^2+^, Mg^2+^) by liquid ion chromatography (Dionex ICS 1100), while the other quartz filter was used to quantify the total carbon (TC), organic carbon (OC), and elemental carbon (EC) with a Dual-Optical Carbonaceous Analyzer (Sunset Laboratory, Tigard, OR, USA), as reported in [33]. The sampling line to assess TC was equipped with an additional backup quartz filter (quartz behind quartz filter, QBQ) [34,35] to account for the positive artefact caused by volatile compounds. A quarter of each quartz filter intended for the ions analyses was recovered and used for the analysis of PAHs associated with the exhaust (Supplementary materials and methods and Appendix B Table A2).

### 2.3. Emission Index Determination

The emission indexes from the fossil fuel and the biofuel blend were calculated for particle number, TNMHC, NO_2_, SO_2_, OC, and EC, accounting for interference factors that may have affected the dilution of the emissions at the exit of the turbofan, such as: the differences in meteorological variables (wind speed and wind direction), the different volume of the cone of emissions under different thrusts, and the slightly different alignment between the sampling line inlet and the emission cone axis. 

The emission index (*EI*) represents the mass of emitted pollutant as a function of the mass of consumed fuel. To calculate the emission indexes for each pollutant, we assumed that all the mass of fuel was completely burnt during the test and that the measured CO_2_ was apportionable only to the fuel burnt and its dilution in the atmosphere (*FDpr*), in accordance with [36,37].

(1) $$FD_{pr}=\left(\frac{EI_{CO2}}{CO2_{pr}}\times C_{pr}\right)\times \frac{1}{V_{r}}\;$$

where *p* stands for the specific test, *r* stands for the RPM (thrust selected), *EI_CO2_* represents the total emission of CO_2_, *CO*2*_pr_* is the mean CO_2_ concentration with the background value subtracted, while *C_pr_* is the kilograms of fuel used in each test and under the selected thrust (after each test, the mass of fuel remaining in the AMX jet tanks was weighed), and, finally, *V_r_* is the dilution volume for each RPM calculated according to Equation (2):

(2) $$V_{r}=\left[\overset{n}{\underset{p=1}{\sum }}\left(\frac{EI_{CO2}}{CO2_{pr}}\times C_{pr}\right)\right]\times \frac{1}{n}\;with\;n=5;r=50\%\;or\;70\%\;$$

For the offline measurements, the subsequent correction factors were applied to account for different dilutions of the emissions:

(3) $$FD_{p}=\left(\frac{EI_{CO2}}{CO2_{p}}\times C_{p}\right)\times \frac{1}{V}$$

where *EI*_*CO*2_ is the total emission of CO_2_, and *CO*2*_p_* is the measured carbon dioxide during a test *p* (for the filters, the samples of different thrust settings were pooled), while *C_p_* is the mass of fuel used during a test *p*, and *V* is the dilution volume calculated according to Equation (4):

(4) $$V=\left[\overset{n}{\underset{p=1}{\sum }}\left(\frac{EI_{CO2}}{CO2_{p}}\times C_{p}\right)\right]\times \frac{1}{n}\;with\;n=5\;$$

The emission index for the different parameters (*EI_x_*) was then calculated according to [38] and Equation (5):

(5) $$EI_{x}=\frac{x_{fd}\times S\left(x_{fd}\right)}{CO2_{fd}}\times EI_{CO2}\;$$

where *EI_CO_*_2_ is the total mission index for CO_2_ according to (1), *X_fd_* and *CO*2*_fd_* are the mass concentrations of the parameter *x* and of CO_2_ with their background levels subtracted, while *S*(*_Xfd_*) is a dimensional conversion factor.

### 2.4. Cell Culture and Exposure

Cell culture: Human bronchial epithelial cells (BEAS-2B, ECACC, Sigma-Aldrich, St. Louis, MI, USA) were maintained in LHC-9 medium (Thermo Fisher Scientific, Waltham, MA, USA) at 37 °C and 5% of CO_2_. 

One week before the exposure, cells were cultured at a density of 45.000 cells on the apical side of well inserts (Corning, 0.4 µm pore diameter, collagen-coated). The LHC-9 medium was changed every two days in both the apical and basolateral compartments. The day before the exposure, the apical medium was removed from all the inserts and cells were allowed to differentiate for 24 h (more details on the protocol in [39]). 

Exposure condition: Online exposures to combustion exhaust of fossil fuel and biofuel blends were performed by means of two Cultex RFS modules. A sampling line, with a cyclone to select particles with an aerodynamic diameter smaller than 2.5 µm, was placed into the expansion chamber and directly connected to the exposure modules. In each module, three inserts were exposed directly to the combustion emissions, and three inserts were exposed to filtered air (for more detail, see [39]) and used as controls. At the end of the exposure, the cells and the medium in the basolateral compartment were recovered and directly manipulated according to the relative biological endpoints selected; for two experiments with the biofuel blend, a recovery of one hour was allowed, placing the cells into an incubator at 37 °C and 5% of CO_2_ just after the end of the exposure. Exposure doses were calculated considering the number particle concentration measured by the SMPS and the OPC, these instruments being connected to the same sampling line of the exposure modules. For the toxicological test, exposure lasted for the complete duration (around 45 min) of each single emission test; therefore, emissions were collected at both 50 and 70% of thrust. 

### 2.5. Toxicological Evaluation

Cell viability was assessed by measuring the LDH activity in the media recovered from control and exposed cells. Briefly, the recovered medium was diluted 1:1 *v*/*v* with CytoTox-One reagent (CytoTox-ONE™ Homogeneous Membrane Integrity Assay Promega, Madison, WI USA) and incubated for 10 min. The emissions of the conversion of the non-fluorescent resazurin to the fluorescent resorufin was measured in a fluorimeter (excitation λ 560 nm, emission λ 590 nm, Glomax Discover System, Promega, Madison, WI USA).

Inflammatory cytokines (IL-6, IL-8, IL-1β, IL10, TNF-α, and IL-12) released in the medium underneath the cells were measured using the Human Inflammatory Cytokine CBA (Becton Dickinson, Franklin Lakes, NJ, USA) according to the manufacturer’s instructions. Briefly, the medium from exposed and control cells, or the standards for the calibration curves, were mixed with the reaction beads and the antibodies stained with fluorescent phycoerythrin. After 3 h of incubation, the samples were read with a FACS Calibur (Becton Dickinson, Franklin Lakes, NJ, USA) and the pg/mL of the different cytokines determined according to the calibration curves. 

Gene expression was assessed by extracting total RNA with the Quick-RNA Microprep Kit (Zymo Research, Irvine, CA, USA), according to the manufacturer’s instructions. The amount and purity of the extracted RNA were evaluated using a fiber-optic Nanodrop ND-1000 spectrophotometer (Thermo Fisher Scientific, Waltham, MA, USA), calculating the 230/260 and 260/280 absorbance ratios. First-strand cDNA was synthesized using a high-capacity RNA-to-cDNA kit (Thermo Fisher Scientific, Waltham, MA, USA) according to the manufacturer’s instructions. Analysis of the gene expression was carried out with quantitative real-time PCR (qRT-PCR) using SYBR Green master mix (Thermo Fisher Scientific, USA) and the following primers, as previously described: CYP1B1 [40], NQO1 [41], HO1 [42], IL-6 [43], and 18s [27]. qRT-PCR was performed on a StepOnePlus thermocycler (Thermo Fisher Scientific, USA) using the thermal cycling conditions consisting of a holding stage at 50 °C for 2 min and 95 °C for 10 min, followed by 40 cycles of each PCR step: (denaturation) 95 °C for 15 s and (annealing/extension) 60 °C for 1 min. 

The relative fold change 2^−ΔΔCT^ method was used to determine the relative quantitative gene expression compared with 18s as endogenous controls.

All PCR reactions were performed in triplicate and data were expressed as the mean ± standard error (SE).

### 2.6. Human Exposure Assessment

Particle size number distributions from the different tests were used to quantify the potential maximal human lung-deposited dose with the MPPD 3.04 inhalation model. Five minutes of data were integrated into each test to obtain a mean particle number concentration. These mean values were used to calculate the deposition of the particles in the trachea–bronchial and lung regions according to [39]. The model was run considering a 60 percent stochastic lung with a functional residual capacity of 3.3 L and an URT volume of 50 mL. Aerosol density was considered constant over the different size ranges and equal to 1 g/cm^3^ [44], and all the particles were modelled as perfect spheres. The variable exposure condition option was run considering the duration of each measurement as the time of exposure, nasal respiration, a tidal volume of 1250 mL, a breathing frequency of 20 inspirations per minute, and an inspiratory fraction of 0.5 with no pause. Deposition only, without clearance, was considered to obtain the integrated total mass deposited for each average diameter. Deposited doses in µg/cm^2^ of the pulmonary or trachea–bronchial surfaces were calculated according to [45]. The reported calculations considered the emissions during the whole duration of each test, and therefore without separating emissions at 50% and 70% of thrust, to allow for comparison with the toxicological outcomes. 

### 2.7. Statistical Analyses

Emission factors are reported as average and quadratic error to provide relevant intervals. Chemical characterization results are reported as mean and standard error. Biological results are reported as mean and standard deviation, and statistical significance was tested with ANOVA followed by post hoc analysis.

## 3. Results

### 3.1. Emission Characterization: Online Monitors

Volatile emissions are reported (Table 2) in terms of TNMHC, SO_2_, NO_2_, and CO_2_ (µg or mg per m^3^), with the background levels already subtracted (Appendix B Table A3). Average emission concentrations (with the relative standard error) are reported for each fuel and for each thrust (RPM) tested. Higher TNMHC was observed during lower thrusts with a slightly higher, but not significant, increase in biofuel blends vs. the fossil fuel. NO_2_, SO_2_, and CO_2_ emissions increased with the thrust applied, as expected from a better combustion efficiency. All gases showed a higher concentration during the biofuel combustions, but surprisingly higher were the values determined for SO_2_, which were almost an order of magnitude higher. Notably, this difference is related to the sulfur content of the fossil fuel used to prepare the biofuel blends (Appendix B Table A1) and not to a direct effect of the biofuel per se. 

The emissions of quasi-ultrafine (PM_0.3_) and fine (PM_0.3–1.7_) particles are reported, including the number particle concentration and relative mean geometric diameter for each fuel and thrust tested (Table 3), with the background effect already subtracted (the latter is reported in Appendix B Table A4). The data show that the emission of particles increases (for PM_0.3_ and PM_0.3–1.7_) with the thrust applied and that this higher emission is related to particles with a lower geometric mean diameter (GMD).

Similar increases were evident also for OC, EC, and TC (Table 4). Interestingly, the ratio of EC/OC was much higher for the fossil fuel at the lower thrust compared to the biofuel blends. The latter showed a lower ratio between EC and OC (always below 1), suggesting a remaining fraction of partially non-combusted organic species, in agreement with the TNMHC values. Background levels are reported in Appendix B Table A5 and Table A6.

### 3.2. Emission Characterization of Airborne Pollutants: Offline Analyses

Besides the online measurements, which allowed us to discriminate between different thrusts, offline filter analyses (sampling the emissions at different thrusts for each experiment) were carried out to characterize the emissions of selected chemical species. Here, we report the data for the ionic species and the metal and trace element species (data on PAHs, EC, and OC measured on the sampled filters are reported in Appendix B Table A2 and Table A6, respectively). Airborne mass concentrations of the ionic species (Table 5) showed higher values for nitrate (NO_3_^−^) and nitrite (NO_2_^−^) during fossil fuel combustion compared to the biofuel blends that, on the contrary, were characterized by higher concentrations of sulfate (SO_4_^2−^). 

The concentrations of the ionic species were also measured in the QBQ filters (Table 6), which showed that these chemical species partitioned between the two filters during the sampling. In this case, the differences observed between the two fuels are less evident, and a slight increase in the average content of nitrite in the biofuel samples (15.00 + 0.34) compared to the fossil fuel (9.80 + 0.25) was measured.

The metal and elemental species determined by XRF showed similar concentrations between the fossil fuel and biofuel blends during the different replica. A relative increase in the concentration of antimony and barium was evident in the fossil fuel emission sample, but this increase was not significant (Appendix B Table A7).

### 3.3. Emission Indexes

The possibility to compare the relative emissions of the two fuels (fossil fuel vs. biofuel blends) was also explored by determining the specific emission indexes per unit of fuel burnt during the tests (Table 7). The emission indexes per unit of fuel (Kg) showed that the biofuel blends were higher emitters of particles in the ultrafine mode (a diameter below 100 nm) and partly in the accumulation mode (roughly, the diameter was between 100 and 300 nm). Biofuels also increased the release of NO_2_ and OC, and the other parameters were comparable among the two fuels. Again, the SO_2_ higher emission index should not be considered as representative of the biofuel blends but as an artefact due to the properties of the fossil fuel used to prepare the blends. Similar results were obtained from the sampling on filters (Appendix B Table A8) which also showed a reduced emission index for biofuel blends when considering nitrite and nitrate emissions and for the metal and trace elements detected. 

### 3.4. Toxicological Effects

Direct exposure to the airborne emissions from the differential combustion of the fossil fuel and biofuel blends aimed at determining whether, besides possible chemical and physical differences among the emissions from the two fuels, contrasting effects were accountable when focusing on the acute responses of the lung epithelia. 

The exposure doses calculated for the in vitro models (Table 8) showed higher maximal deposition during the experiments with the biofuel blends. The third experiment with the biofuel blend showed the highest exposure doses compared to the other fuels. Interestingly, biofuel blend exposures were characterized by a higher contribution of deposited UFP mass compared to the fossil fuel ones, which accounted for the higher number of these particles emitted during the biofuel tests. 

**Figure 2:**
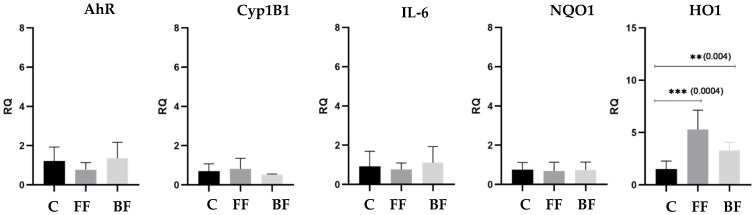
Differential gene expression after exposure to filtered air (C), fossil fuel emissions (FF), and biofuel blend emissions (BF).

**Figure A1:**
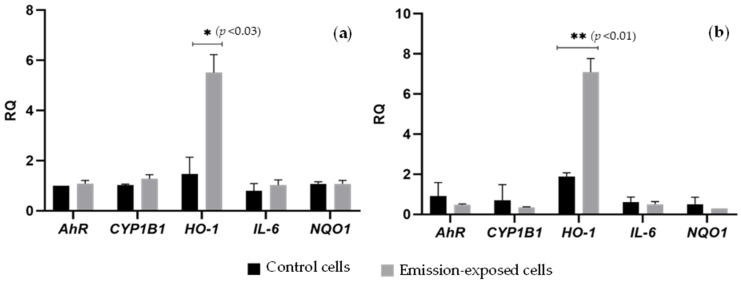
Gene expression of selected genes during the two exposures to fossil fuel emissions. Fossil I (**a**) and fossil II (**b**) emissions caused similar gene expression variation. All the other genes were not modified.

**Figure A2:**
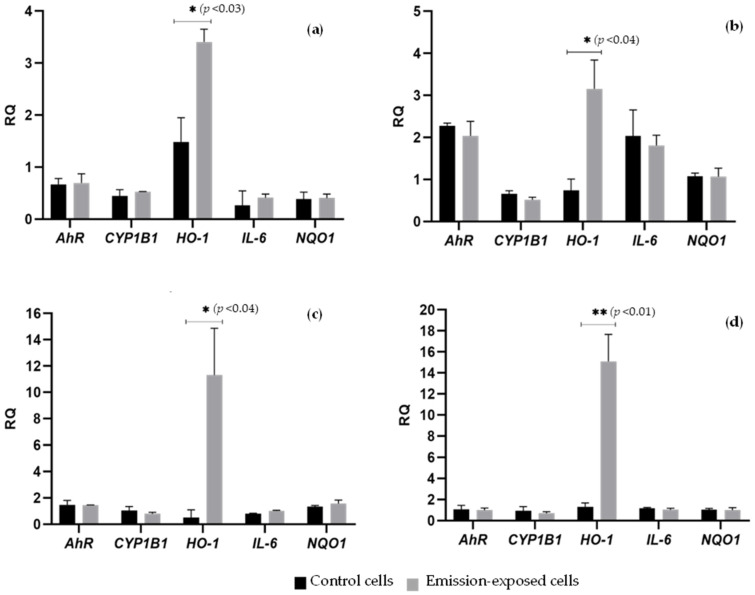
Gene expression of selected genes during the different biofuel blend emission tests. Biofuels I and III gene expression were analyzed just after the end of the tests (**a**,**b**), while for biofuels II and IV the cells were left to recover for 1 h after the end of the exposures (**c**,**d**). The recovery period dramatically increased the expression of the antioxidant HO1 gene. All the other genes were not modified.

Cell viability showed no significant differences among the treated and control groups (data not shown). The cytokine panel, characterized in the collected cell media, showed that no quantifiable proteins were detectable right after the exposure; only after 1 h of recovery from the exposure was the IL-6 quantified in the medium underneath the cells, but no statistical difference was reported between the control (13.8 pg/mL + 4.6 pg/mL) and exposed cells (12.4 pg/mL + 1.4 pg/mL, biofuels after 1 h recovery). The analysis of the expression of a set of selected genes also showed slight (not statistically significant) changes between the groups, except for the HO1 gene (Figure 2). By grouping all the samples, HO1 was statistically increased after the exposure to both the fossil fuel and the bio-blend emissions compared to the control cells, with the highest gene expression increase demonstrated in response to the fossil fuel exposures. In fact, both of the exposures with the fossil fuel significantly increased the expression of HO1 (Appendix B Figure A1), while minor non-significant variations were observed for all other genes. In comparison, the exposure to biofuel blend emissions (Appendix B Figure A2, panel a,b) showed a significant gene expression increment but with a lower magnitude. Interestingly, after 1 h recovery, the relative increase in HO1 expression in biofuel-exposed cells (Appendix B Figure A2, panel c,d) increased dramatically, thus suggesting that the oxidative effects triggered by the emissions drive an acute and significant effect on human lung cells.

### 3.5. Human Exposure

**Figure 3:**
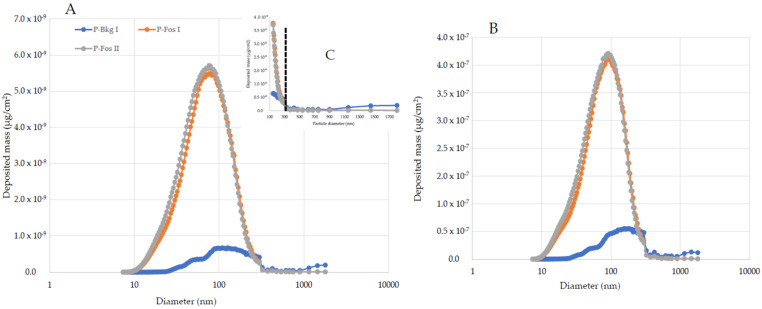
Pulmonary deposition of fossil fuel emissions (P-Fos I and P-Fos II) emitted, and the background (P-Bkg I) particles for the pulmonary (**A**) and trachea–bronchial (**B**) regions. Maximal contribution of emission is within 300 nm (**C**), after which background particulate distribution accounts for the measured deposition. One maximal peak of deposition (80 nm) is evident.

**Figure 4:**
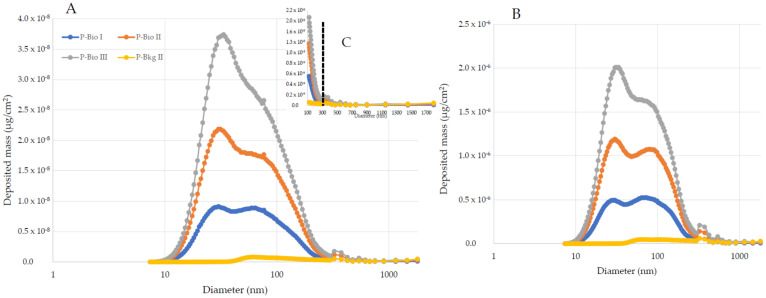
Pulmonary deposition of biofuel blends emitted (P-Bio I to III), and the background (P-Bkg II) particles for the pulmonary (**A**) and trachea–bronchial (**B**) regions. Maximal contribution of emission is within 300 nm (**C**), after which background particulate distribution accounts for the measured deposition. Two peaks of deposition are reported, one at 30 nm and one at around 80 nm.

Potential human exposure calculated with the MPPD model showed (Table 9) higher lung-deposited doses during the biofuel tests. Interestingly, the size-resolved depositions (Figure 3 and Figure 4) of the two sets of experiments showed some differences. Fossil fuel deposition was dominated by a main peak of particles with a diameter of around 80 nm, while biofuel blends were characterized by a distribution with two peaks, the first referring to particles with a diameter of around 30 nm and the second around 80 nm. All the experiments showed depositions well above the background level within 300 nm of particle diameter. After this, the background particles were affecting the deposition doses in all the experiments, suggesting that the emissions from the jet were characterized by particles below 300 nm.

## 4. Discussion

Reducing the impact of anthropogenic activities on climate change is the most important environmental issue if the present generations want to maintain the equilibrium of the several processes that allow life on earth [46].

The aviation sector impacts climate change due to its massive use of fossil fuels [47,48] and the steadily increasing number of flights recorded in the last few decades [9]. In recent years, several research groups have reported data on the potential benefit of reducing the use of fossil fuels by substitution with biofuel blends. Dray et al. [48] reported a significant benefit in terms of CO_2_ equivalent [49] by substituting the fuels of fossil origin with cellulosic biomass-derived fuels. More recently, Staples et al. [50] enlarged the study comprising different feedstock sources to produce alternative aviation fuels, providing additional evidence for a potential reduction in GHG emissions from the aviation sector. In addition, the use of biofuel has been shown to reduce not only climate-changing emissions but also particulate emissions [44,51,52,53], which in turn might improve the climate by reducing the formation of contrail cloudiness [54]. Our results are in line with these previous results and provide additional evidence for the emission factors and the potential health impact of emissions from a jet fueled with a reference fossil fuel and different blends of biofuel and the fossil fuel. In fact, the reduction in volatile organic compounds (TNMHC) and elemental carbon (EC) is in line with previous results [18,22,55]. On the contrary, other parameters (fine and ultrafine PM number concentration, OC, and NO_2_) show a surprising increase, although similar emission factors have been reported for commercial flights [56]. These data are, however, related to the chemical properties of the Jet A-1 fossil fuels used (Appendix B Table A1) rather than to the biofuel blends. The relevance of sulfur in modifying the emission factors agrees with the factors reported by [20] and [57,58] that showed the particles’ emission indexes varying by two orders of magnitude, with higher emission values affected by the higher sulfur content.

In addition, the variation in the ratio between EC and OC that we also report here (Table 3) agrees with the data from Moore and co-workers [20] that showed a reduction in the emission of black carbon at a higher fuel flow rate in the jet engine, while the emission of organic compounds was less related to the fuel flow rate. Our data, accordingly, show that at the highest thrust tested (almost 95%) the concentration of EC in µgC/m^3^ is around half of the concentrations measured at lower thrusts. 

Ultrafine particles are a major environmental concern in areas close to major airports [24,25]. The values recorded and here reported are in perfect agreement with those reported by [59]. In fact, the authors report occupational exposures to ultrafine particles as high as 10^6^–10^7^ particles per cm^3^. Notably, Michaelis et al. [60] recently showed that values for the in-cabin measurement of UFP can vary in the order of 10^4^ to 10^5^ particles/cm^3^. Ren et al. [61] demonstrated that the in-cabin values were highly related to the take-off operations in airplanes waiting, before departure, in areas downwind the take-off area.

The potential impact of aviation emissions on human health is in fact a subject of great debate [13]. Berret et al. [62] reported a significant impact of aviation emissions on mortality. Cavallo et al. [63] outlined the potential genotoxic effects of airport emissions in exposed workers, while the potential formation of ozone from aircraft emissions has been recently related to mortality and skin cancer [64]. Here, we showed that biofuel has a lower capability to induce acute epithelial lung response (lower expression of the oxidative protection gene, HO1) compared to fossil fuel. Notably, the recovery of exposed cells for just one hour induced a dramatic increase in HO1 expression. Nonetheless, the increase in the heme-oxygenase gene reported here is in line with a recent paper [65,66] suggesting that the activation of this gene protects against the adverse effects of air pollution. Interestingly, the activation of this gene has also been described by [31] after 24 h of exposure to airplane emissions at 85% or ground-idle thrusts (controlled using the engine combustor inlet temperature and not, as in our experiments, by actually increasing the airplane thrust) as showing a reduction in biofuel (32% *v*/*v* HEFA/Jet A-1) HO1 induction. Moreover, 90 days of exposure of mice to airport samples was reported to induce significant lung damage [67].

The lung-deposited mass doses, considering the short time of exposure defined by the experimental approach, agree with the expected particle deposition reported by other authors (summarized in [68]) and during combustion events such as forest burning [69]. 

In conclusion, we report novel data on the possibility of using biofuels to improve sustainability in the aviation sector: biofuel blends should therefore be considered to reduce CO_2_ emissions from the aviation sector. Our emission factors clearly showed that a refinement of the Jet A-1 requirements may be relevant for the reduction in SO_2_. 

The data agree with those previously reported and add a first preliminary evaluation of the hazard of aircraft emissions and the expected lung doses. These data improve our understanding of the exposure doses of people living in proximity of, or working in, an airport and also underline the importance of assessing the health impact of biofuel blends, at least on the respiratory system. In fact, our data show that acute exposure to fossil fuel and biofuel blends might cause an oxidative burst in lung tissue and that the expected deposited doses in human lungs increase with the content of the biofuel in the blend. Significantly, biofuel determines the deposition of ultrafine particles of 30 nm. 

Significant improvement in the sustainability of the aviation sector may be obtained by biofuels, possibly reducing at the same time the potential health effects on exposed populations, although additional investigation should be devoted to better understand the impacts on health of biofuel blend emissions, possibly considering sub-chronic exposures and the use of biological models representative of other potential target tissues.

## Figures and Tables

**Table 1:** Specification of the Spey Mk 202 engine model used to test the different fuels.

Property	Prop. Info	Components	Comp. Info	Performance	Perf. Info
Type	Low-bypass turbofan	Compressor	Axial flow, 5-stage LP, 12-stage HP	Maximum thrust	54 kN, with reheat 91.2 kN
Length	5.2 m	Combustors	10 can-annular combustion chambers	Air mass flow	92.53 kg/s
Diameter	1.1 m			Specific fuel consumption	55.2 (g/kN)s with after burner, 17.8 (g/kN)s at military thrust
Dry weight	1856 kg	Turbine	2-stage LP, 2-stage HP	Thrust-to-weight ratio	5:1

**Table 2:** Emission concentrations (reported as mean with the relative standard error) for volatile compounds measured during the combustion tests after accounting for the dilution factor and background effect.

Fuel	Replica	RPM	TNMHC	Std.err	SO_2_	Std.err	NO_2_	Std.err	CO_2_	Std.err
		%	mg/m^3^	mg/m^3^	µg/m^3^	µg/m^3^	µg/m^3^	µg/m^3^	mg/m^3^	mg/m^3^
Fossil	I	50	10.6	3.4	8.1	2.2	332	61	585	92
	I	70	9.8	4.7	14.9	1.4	710	146	1002	84
	II	50	10.2	2.4	5.6	1.8	316	54	478	124
	II	70	9.1	2.9	12.4	2.5	745	228	1057	219
Biofuel	I	50	10.9	2.5	105.7	9.1	467	100	687	212
	I	70	10.5	3.1	256.0	76.8	1231	210	1465	201
	II	50	16.9	3.2	209.5	60.0	697	278	788	293
	II	70	10.6	4.2	459.9	75.4	1521	190	1740	157
	III	50	19.4	4.5	406.0	86.5	826	175	1069	351
	III	70	13.7	4.9	662.6	72.8	1861	166	2004	121
	IV	60	12.0	3.3	234.6	41.9	519	204	621	142
	IV	95	7.7	1.6	455.5	68.6	1108	127	1334	139

**Table 3:** Emissions for the fossil fuel and biofuel blends in terms of number of quasi-ultrafine (PM_0.3_) and fine (PM_0.3–1.7_) particles and the geometric mean diameter (GMD) after accounting for the dilution factor and background influence.

Fuel	Replica	RPM	PM_0.3_	Std.err	GMD	Std.err	PM_0.3–1.7_	Std.err
		%	#/cm^3^	#/cm^3^	nm	nm	#/cm^3^	#/cm^3^
Fossil	I	50	1.03 × 10^6^	3.20 × 10^5^	29.1	1.4	14.2	2.8
	I	70	1.66 × 10^6^	2.47 × 10^5^	23.3	1.5	23.1	5.5
	II	50	1.17 × 10^6^	2.01 × 10^5^	28.7	1.1	13.5	2.4
	II	70	2.00 × 10^6^	3.00 × 10^5^	22.9	2.4	25.8	10.6
Biofuel	I	50	2.62 × 10^6^	1.93 × 10^5^	24.0	1.2	13.0	2.9
	I	70	6.13 × 10^6^	8.14 × 10^5^	21.5	0.6	35.3	7.1
	II	50	3.54 × 10^6^	3.37 × 10^5^	22.6	1.0	22.0	5.7
	II	70	6.43 × 10^6^	2.52 × 10^6^	21.5	2.0	48.2	11.3
	III	50	5.24 × 10^6^	7.70 × 10^5^	23.4	1.0	23.7	5.6
	III	70	8.60 × 10^6^	2.88 × 10^5^	23.8	0.3	67.8	14.3
	IV	60	2.89 × 10^6^	5.12 × 10^5^	22.3	1.8	29.1	6.5
	IV	95	6.87 × 10^6^	5.69 × 10^5^	21.7	0.6	41.8	11.4

**Table 4:** Emission concentrations for OC, EC, and TC measured during the different tests—already accounting for the dilution factors and background effect—and the EC/OC ratio.

Fuel	Replica	RPM	OC	Std.err	EC	Std.err	TC	Std.err	EC/OC
		%	µgC/m^3^	±µgC/m^3^	µgC/m^3^	±µgC/m^3^	µgC/m^3^	±µgC/m^3^	
Fossil	I	50	28.6	6.9	48.1	6.9	76.7	20.3	1.68
	I	70	35.4	11.0	25.3	11.6	60.7	34.9	0.71
	II	50	24.9	8.3	59.1	7.4	106.3	31.8	2.37
	II	70	37.6	10.4	26.3	13.8	63.9	39.6	0.70
Biofuel	I	50	64.7	8.7	52.7	7.9	117.4	23.9	0.81
	I	70	102.4	11.8	66.2	8.9	168.6	30.0	0.65
	II	50	50.8	6.5	39.9	6.6	90.6	19.4	0.79
	II	70	126.6	14.6	66.4	11.6	193.0	40.7	0.52
	III	50	148.7	12.0	66.0	8.1	214.6	31.7	0.44
	III	70	154.6	17.7	52.1	10.8	206.7	49.3	0.34
	IV	60	101.3	9.8	37.2	6.2	138.5	27.4	0.37
	IV	95	95.2	13.4	32.9	8.4	128.2	38.5	0.35

**Table 5:** Airborne concentration of ionic species measured on filters collected during the different tests. Data are reported as average and standard error (n > 2). Concentrations below the detection limit of the technique are reported in the table as nd (not detected). Blank cells represent missing values.

Fuel	Replica	NO_2_^−^	Std.err	NO_3_^−^	Std.err	SO_4_^2−^	Std.err	NH_4_^+^	Std.err
		µg/m^3^	±µg/m^3^	µg/m^3^	±µg/m^3^	µg/m^3^	±µg/m^3^	µg/m^3^	±µg/m^3^
Fossil	I	36.0	1.0	7.5	0.4	0.35	0.01	nd	nd
	II					0.42	0.03	nd	nd
Biofuel	I	6.1	0.6			1.0	0.2	nd	nd
	II					1.9	0.3	0.43	0.03
	III					1.5	0.2	0.38	0.02
	IV			2.1	0.5	3.5	0.4	1.48	0.05
Background		0.114	0.003	0.71	0.08	0.79	0.04		

**Table 6:** Ionic species concentration in the QBQ filters. Data are reported as average (n > 2) and standard error. Concentrations below the detection limit of the technique are reported in the table as nd (not detected).

Fuel	Replica	NO_2_^−^	Std.err	NO_3_^−^	Std.err	SO_4_^2−^	Std.err	NH_4_^+^	Std.err
		µg/m^3^	±µg/m^3^	µg/m^3^	±µg/m^3^	µg/m^3^	±µg/m^3^	µg/m^3^	±µg/m^3^
Fossil	I	15.8	0.3	7.8	0.4	17	0.4	nd	nd
	II	3.8	0.2	7	1	18	1	nd	nd
Biofuel	I	2.12	0.09	7	1	17	2	nd	nd
	II	13.7	0.3	9	1	18	0.4	nd	nd
	III	33.2	0.7	17.9	0.4	21	0.5	nd	nd
	IV	11.0	0.3	5.9	0.3	13	0.3	nd	nd
Background		7.34	0.2	4.3	0.2	8.7	0.7	nd	nd

**Table 7:** Emission indexes calculated per kg of fuel consumed during each test, grouping the experiments with thrust equal to 50% and with thrust equal to 70%.

	Biofuel Blend		Fossil Fuel		Ratio Bio/Fossil	
	50%	70%	50%	70%	50%	70%
	Average (quadratic error)	Average (quadratic error)	Average (quadratic error)	Average (quadratic error)	Average (quadratic error)	Average (quadratic error)
Total particle number D_p_ > 7nm (#/Kg)	1.4 × 10^16^ (2.6 × 10^15^)	1.4 × 10^16^ (2.6 × 10^15^)	6.4 × 10^15^ (2.3 × 10^15^)	5.6 × 10^15^ (1.2 × 10^15^)	2.2	2.3
Total nanoparticles D_p_ < 40 nm (#/Kg)	1.2 × 10^16^ (2.2 × 10^15^)	1.2 × 10^16^ (2.2 × 10^15^)	4.5 × 10^15^ (1.6 × 10^15^)	4.5 × 10^15^ (1.2 × 10^15^)	2.6	2.6
Total ultrafine particles 40 < D_p_<100 nm (#/Kg)	2.1 × 10^15^ (7.6 × 10^14^)	2.1 × 10^15^ (7.6 × 10^14^)	1.6 × 10^15^ (6.8 × 10^14^)	9.1 × 10^14^ (2.7 × 10^14^)	1.4	1.1
Accumulation-mode particles 100 < D_p_ < 300 nm (#/Kg)	1.4 × 10^14^ (4.6 × 10^13^)	1.4 × 10^14^ (4.6 × 10^13^)	1.2 × 10^14^ (3.5 × 10^13^)	1.0 × 10^14^ (4.5 × 10^13^)	1.1	0.7
PM_0.3_, D_p_ > 300 nm (#/Kg)	7.3 × 10^10^ (5.8 × 10^10^)	7.3 × 10^10^ (5.8 × 10^10^)	8.0 × 10^10^ (4.4 × 10^10^)	7.5 × 10^10^ (3.0 × 10^10^)	0.9	1.2
TNMHC (g/Kg)	58.5 (22.0)	58.5 (22.0)	60.1 (23.8)	28.9 (16.7)	1	0.7
NO_2_ (mg/Kg)	2460.4 (1229.2)	2460.4 (1229.2)	1867.5 (470.5)	2225.0 (820.4)	1.3	1.3
SO_2_ (mg/Kg)	842.4 (309.4)	842.4 (309.4)	39.1 (16.4)	41.9 (9.3)	21.6	19.3
OC (mgC/Kg)	313.6 (63.7)	313.6 (63.7)	153.7 (63.2)	111.7 (46.3)	2.0	2.1
EC (mgC/Kg)	199.1 (50.8)	115.2 (34.8)	312.8 (59.7)	78.9 (55.0)	0.6	1.5

**Table 8:** In vitro model doses of exposure. Doses are reported as number of particles and mass of particles deposited per square cm of cell surface. The relative contribution of ultrafine particles (UFP, particles with diameter < 100 nm) is also reported.

		Exposure Dose (#/cm^2^)			Exposure Dose (µg/cm^2^)		
Fuel	Replica	Total PM Dose	UFP Dose	UFP/PM	Total PM Dose	UFP Dose	UFP/PM
	I	1.38 × 10^5^	1.38 × 10^5^	0.995	2.47 × 10^−6^	1.75 × 10^−6^	0.709
Fossil	II	1.68 × 10^5^	1.67 × 10^5^	0.996	2.66 × 10^−6^	1.96 × 10^−6^	0.738
	I	5.38 × 10^5^	5.37 × 10^5^	0.998	5.21 × 10^−6^	4.31 × 10^−6^	0.827
Biofuel	II	6.14 × 10^5^	6.14 × 10^5^	0.998	5.85 × 10^−6^	4.91 × 10^−6^	0.840
	III	6.79 × 10^5^	6.78 × 10^5^	0.998	7.46 × 10^−6^	6.52 × 10^−6^	0.873
	VI	6.30 × 10^5^	6.30 × 10^5^	0.999	5.00 × 10^−6^	4.46 × 10^−6^	0.892

**Table 9:** Deposited doses of particles during emission tests and during background monitoring. Data are reported as deposited mass (µg/cm^2^) of trachea–bronchial (TB) or pulmonary (P) epithelial surface.

Fuel	Replica	TB Deposition (µg/cm^2^)	P Deposition (µg/cm^2^)
Background	I	2.43 × 10^−6^	3.09 × 10^−8^
Fossil	I	1.61 × 10^−5^	2.41 × 10^−7^
	II	1.69 × 10^−5^	2.57 × 10^−7^
Biofuel	I	3.18 × 10^−5^	5.00 × 10^−7^
	II	6.90 × 10^−5^	1.09 × 10^−6^
	III	1.07 × 10^−4^	1.72 × 10^−6^
Background	II	2.54 × 10^−6^	3.36 × 10^−8^

## Data Availability

Not applicable.

## Appendix A

Supplementary materials and methods for Appendix B Table A2. The quarters of filters were pooled according to the fuel they derived from to obtain two distinct pools, one for the fossil fuel and one for the biofuel. The pools were subjected to an accelerated solvent extraction (ASE 200 Dionex) in a low-volume vial (7 mL) with the following settings: extraction solvent hexane/acetone 1:1, T extraction 120 °C at 1500 psi, two consecutive extraction cycles with 5 min of static phase. Since the extracts were also prepared for subsequent toxicological analysis (not reported here), the filters were not spiked with reference materials to avoid possible contamination or formation of artefacts. At the end of the extraction, circa 10% in weight of the extract was recovered, spiked with deuterated reference compounds (antrhacene-d10, pyrene-d10, crysene-d12, perylene-d12), dried to a final volume of 150 μL, and subjected to GC-MS for PAH quantification.

GC/MS (Agilent 7890a GC/5975c MS) equipped with a capillary column DB-5ms (Agilent, 30 m × 250 μm × 0.25 μm) was run with the following thermal program: 40 °C for two min, 20 °C/min up to 200 °C and then rest for 1 min, 4 °C/min up to 300 °C and then rest for 1min, 20 °C/min to reach 320 °C and then rest for 7 min, ionization at 70 eV, and data acquisition in Single Ion Monitoring (SIM).

## Appendix B

**Table A1:** Chemical and physical characterization of bio-blend fuel 17% and content of sulfur in the bio-blend and fossil fuels used for reference test and blend tests.

Parameter	Limit Values for Jet A-1 Fuel Type	Value	Test Method
	Reference Values		
Color	none	+28	ASTM D 156
Density at 15 °C (Kg/m^3^)	775.0–840.0	793.3	ASTM D4052
Distillation initial point (°C)	none	158	ASTM D86
Distillation initial point (10%) (°C)	≤205	173	ASTM D86
Distillation initial point (50%) (°C)	none	198	ASTM D86
Distillation initial point (50%) (°C)	none	236	ASTM D86
Distillation final point (°C)	≤300	262	ASTM D86
Distillation residual (% volume)	≤1.5	1.0	ASTM D86
Distillation losses (% volume)	≤1.5	0.8	ASTM D86
Viscosity at 20 °C (eSt)	≤8.0	3.9	ASTM D445
Flash point (°C)	≥38	45	ASTM D56
Thermal stability at 260 °C: pressure drop (mmHg)	≤25	0	ASTM D3241
Thermal stability at 260 °C: deposit thickness (nm)	≤85	5	ASTDM 3241
Aromatic hydrocarbons (mL/100mL)	≤25.0	16.7	ASTM D1319
Doctor test	negative	negative	ASTM D4952
Corrosion on copper lamina (n)	≤1	1	ASTM D130
Lower calorific value (MJ/Kg)	≥42.80	43.20	ASTM D3338
Smoke point (mm)	≥19	25	ASTM D1322
Total acidity (mg KOH/g)	≥0.015	<0.015	ASTM D3242
Electric conductibility (pS/m)	50–600	90	ASTM D2624
*TOTAL SULFUR CONTENT:*			
Total sulfur in the bio-blend 20% (g/100 g)	≤0.3	0.0928	ASTM D4294
Total sulfur in the fossil fuel used for the blend (g/100 g)	≤0.3	0.1175	ASTM D2622
Total sulfur in HEFA fuel (g/100 g)	≤0.0015	0.0012	ASTM D2622
Total sulfur in the fossil fuel used for the first two reference tests (g/100 g)	≤0.3	0.07	ASTM D4294

**Table A2:** Airborne concentration of PAHs measured on quartz filters. Values are reported as average and standard deviation pooling the whole filter obtained during the fossil fuel and biofuel tests.

PAH	Fossil Fuel		Biofuel Blend	
	ng/m^3^	±ng/m^3^	ng/m^3^	±ng/m^3^
Phenanthrene	60.5	6.1	28.9	2.9
Anthracene	6.3	0.6	6.1	0.6
Fluoranthene	109.5	10.9	121.4	12.1
Pyrene	84.8	8.5	146.7	14.7
Benzo[a]anthracene	92.3	9.2	54.6	5.5
Chrysene	145.2	14.5	116.4	11.6
Benzo[b+j]fluoranthene	363.6	36.4	314.0	31.4
Benzo[k]fluoranthene	152.0	15.2	126.2	12.6
Benzo[a]pyrene	324.8	32.5	277.0	27.7
Indeno[1,2,3-cd]pyrene	407.4	40.7	323.3	32.3
Dibenzo[a,h]anthracene	17.0	1.7	16.3	1.6
Benzo[ghi]perylene	473.6	47.4	415.5	41.6

**Table A3:** Volatile compounds’ emission indexes (in unit mass per cubic meter) measured during background monitoring.

Fuel	TNMHC	Std	SO_2_	Std	NO_2_	Std	CO_2_	Std
	mg/m^3^	mg/m^3^	µg/m^3^	µg/m^3^	µg/m^3^	µg/m^3^	mg/m^3^	mg/m^3^
Fossil	2.3	0.6	6.6	1.1	27	10	1046	139
	2.5	0.4	8.1	0.9	25	6	1015	95
Biofuel	1.9	0.7	6.4	0.7	24	6	998	55
	2.2	0.5	8.9	0.8	19	11	983	54
	2.1	0.7	6.2	1.2	27	7	976	109

**Table A4:** Particulate matter emission indexes (number of particles-#-per cubic cm) measured during background monitoring.

Fuel	Replica	PM_0.3_	std	PM_(0.3–1.7)_	std
		#/cm^3^	#/cm^3^	#/cm^3^	#/cm^3^
Fossil	I	5522.5	741.6	18.4	2.3
	II	5517.8	643.9	14.2	1.6
Biofuel	I	7258.7	3025.7	21.4	2.2
	II	6645.5	2768.5	24.6	2.0
	III	7452.4	2444.7	22.8	3.2
	IV	8873.7	1391.3	37.7	3.8

**Table A5:** Carbon species (OC, EC, and TC) measured during background monitoring. The data are reported as average for the whole fossil fuel and biofuel blend tests. Only biofuel test IV is reported separately.

Fuel	OC	err	EC	err	EC/OC
	µgC/m^3^	± µgC/m^3^	µgC/m^3^	± µgC/m^3^	
Fossil	9.7	1.0	3.4	0.3	0,35
Biofuel	6.9	0.7	1.8	0.2	0,26
Biofuel IV	8.6	0.9	6.5	1.1	0,76

**Table A6:** Concentrations of total PM_2.5_, EC, OC, and total measured PAHs in filters sampled during the different replica. Data are reported as average and the relative error.

Fuel	Replica	PM_2.5_	err	EC	err	OC	err	EC/OC	PAHs	err
		µg/m^3^	±µg/m^3^	µgC/m^3^	±µgC/m^3^	µgC/m^3^	±µgC/m^3^		µg/m^3^	±µg/m^3^
Fossil	I	112.6	7.0	44.0	5.8	25.2	3.5	1.75	2.2	0.2
	II	141.8	8.6	51.4	6.7	36.7	5.0	1.40	2.3	0.2
Biofuel	I	241.0	16.0	69.9	8.7	69.7	9.3	1.00	1.9	0.2
	II	322.8	21.3	64.8	8.2	100.1	16.0	0.65	1.6	0.2
	III	290.2	19.3	65.8	8.4	143.5	19.0	0.46	2.4	0.2
	IV	154.2	8.9	38.4	8.3	65.4	8.7	0.59	1.6	0.2
Background	Fossil	19.5	1.0	3.4	0.3	9.7	1.0	0.35		
	Bio I-III	16.5	1.0	1.8	0.2	6.9	0.7	0.26		
	Bio IV	27.0	1.4	6.5	1.1	8.6	0.9	0.76		

**Table A7:** Concentration of metals during the different tests. Data are reported as average and the relative error.

Fuel	Replica	V		Cr		Ni		Sb		Ba	
		µg/m^3^	err	µg/m^3^	err	µg/m^3^	err	µg/m^3^	err	µg/m^3^	err
Fossil	I	0.0025	0.0004	0.12	0.01	0.08	0.01	0.12	0.02	1.0	0.1
	II	0.0023	0.0004	0.11	0.01	0.021	0.003	0.13	0.03	1.26	0.07
Biofuel	I	0.0051	0.0008	0.11	0.01	0.048	0.008	0.085	0.008	0.29	0.03
	II	0.004	0.001	0.103	0.009			0.08	0.01	0.68	0.09
	III	0.007	0.001	0.126	0.006			0.11	0.02	0.87	0.07
	IV			0.086	0.007			0.042	0.005	0.42	0.03

**Table A8:** Emission indexes obtained from filter samples: data are reported as average for each fuel with the relative quadratic error in brackets.

	Biofuel Blends	Fossil Fuel	Ratio Biofuel vs. Fossil
PM_2.5_ mg/Kg	612.0 (70.4)	455.6 (39.5)	1.3
OC mgC/Kg	219.7 (54.1)	109.9 (21.4)	2.0
EC mgC/Kg	145.6 (31.8)	169.4 (31.5)	0.9
PAHs mg/Kg	4.2 (2.5)	8.0 (3.9)	0.53
NO_2_^−^ mg/Kg	5.1 (0.5)	65 (4)	0.08
HONO mg/Kg	4.8 (0.6)	0.9 (0.1)	5.4
NO_3_^−^ mg/Kg	0 (0)	12.3 (3.6)	-
HNO_3_ mg/Kg	4.2 (0.8)	6.7 (2.4)	0.6
SO_4_^2−^ mg/Kg	3.1 (0.5)	1.4 (0.1)	2.3
H_2_SO_4_ mg/Kg	20 (4)	27 (3)	0.7
NH_4_^+^ mg/Kg	0.80 (0.08)	-	-
V mg/Kg	0.010 (0.001)	0.008 (0.0005)	1.3
Cr mg/Kg	0.12 (0.02)	0.21 (0.03)	0.57
Ni mg/Kg	0.07 (0.02)	0.1 (0.02)	0.7
Sb mg/Kg	0.11 (0.02)	0.28 (0.07)	0.39
Ba mg/Kg	0.4 (0.1)	2.8 (0.7)	0.25
